# Novel *Acinetobacter baumannii* Myovirus TaPaz Encoding Two Tailspike Depolymerases: Characterization and Host-Recognition Strategy

**DOI:** 10.3390/v13060978

**Published:** 2021-05-25

**Authors:** Anastasia S. Shchurova, Mikhail M. Shneider, Nikolay P. Arbatsky, Alexander S. Shashkov, Alexander O. Chizhov, Yuriy P. Skryabin, Yulia V. Mikhaylova, Olga S. Sokolova, Andrey A. Shelenkov, Konstantin A. Miroshnikov, Yuriy A. Knirel, Anastasia V. Popova

**Affiliations:** 1 Moscow Institute of Physics and Technology (National Research University), Institutskiy per. 9, Dolgoprudny, 141700 Moscow Region, Russia; anastasiya.shchurova@phystech.edu; 2 State Research Center for Applied Microbiology and Biotechnology, 24 “Quarter A” Territory, Obolensk, City District Serpukhov, 142279 Moscow Region, Russia; sjurikp@gmail.com; 3 Shemyakin-Ovchinnikov Institute of Bioorganic Chemistry, Miklukho-Maklaya 16/10, 117997 Moscow, Russia; mikhailshneider@gmail.com (M.M.S.); kmi@bk.ru (K.A.M.); 4 N. D. Zelinsky Institute of Organic Chemistry, Russian Academy of Sciences, Leninsky Prospect 47, 119991 Moscow, Russia; Nikolay.Arbatsky@gmail.com (N.P.A.); shash@ioc.ac.ru (A.S.S.); chizhov@ioc.ac.ru (A.O.C.); yknirel@gmail.com (Y.A.K.); 5 Central Scientific Research Institute of Epidemiology, Novogireevskaya 3a, 111123 Moscow, Russia; mihailova@cmd.su (Y.V.M.); shelenkov@cmd.su (A.A.S.); 6 Biology Department, Lomonosov Moscow State University, Leninskie Gory 1, 119234 Moscow, Russia; sokolova@mail.bio.msu.ru; 7 Biology Department, Shenzhen MSU-BIT University, Ruyi Rd. 299, Longgang District, Shenzhen 518172, China

**Keywords:** bacteriophage, *Acinetobacter baumannii*, tailspike depolymerase, glycosidase, capsular polysaccharide, capsular type

## Abstract

*Acinetobacter baumannii*, one of the most significant nosocomial pathogens, is capable of producing structurally diverse capsular polysaccharides (CPSs) which are the primary receptors for *A. baumannii* bacteriophages encoding polysaccharide-degrading enzymes. To date, bacterial viruses specifically infecting *A. baumannii* strains belonging to more than ten various capsular types (K types) were isolated and characterized. In the present study, we investigate the biological properties, genomic organization, and virus–bacterial host interaction strategy of novel myovirus TaPaz isolated on the bacterial lawn of *A. baumannii* strain with a K47 capsular polysaccharide structure. The phage linear double-stranded DNA genome of 93,703 bp contains 178 open reading frames. Genes encoding two different tailspike depolymerases (TSDs) were identified in the phage genome. Recombinant TSDs were purified and tested against the collection of *A. baumannii* strains belonging to 56 different K types. One of the TSDs was demonstrated to be a specific glycosidase that cleaves the K47 CPS by the hydrolytic mechanism.

## 1. Introduction

Hospital-acquired or nosocomial infections occur worldwide despite progress in public health and hospital care [1]. *Acinetobacter baumannii*, a Gram-negative non-fermenting aerobic bacterium, is one of the most significant nosocomial pathogens that causes a wide range of infectious complications, especially in critically ill patients [2]. The spread of multidrug-resistant *A. baumannii* strains presents a serious clinical challenge. In 2017, the World Health Organization set carbapenem-resistant *A. baumannii* as a critical priority target for the research and development of new antimicrobial treatments [3]. 

The application of lytic bacteriophages and enzymes encoded in phage genomes is one of the potential approaches to solve the problem of the spread of antibiotic-resistant *A. baumannii* strains. 

The widespread of *A. baumannii* strains in a hospital environment is facilitated by various factors, such as biofilm formation and the production of capsular polysaccharides (CPSs). CPSs surround the bacterial cells and protect them from external threats [4]. The structures of the CPSs produced by *A. baumannii* are highly diverse, more than 140 capsular types (K types) have been bioinformatically predicted (J.J. Kenyon, Queensland University of Technology, Brisbane, Australia, personal communication), and the CPS structures for more than 40 capsular types (K types) have been biochemically established [5]. CPSs are the primary receptors for *A. baumannii*-phages that carry genes encoding polysaccharide-degrading enzymes or structural depolymerases [5,6,7,8]. Thus, phage depolymerases determine specific interactions of phages with bacterial hosts producing CPS of a certain structure. To date, phages that are specific to K1 (phage P1) [9], K2 (phages vB_AbaP_APK2, φAB6, and vB_AbaP_B3) [5,7,9], K9 (phages vB_AbaP_B1, AM24, BS46) [9,10,11], K19 (phages Fri1 and AS11) [12], K27 (phage AS12) [12], K32 (phage vB_AbaP_APK32) [5], K37 (phage vB_AbaP_APK37) [5], K44 (phage vB_AbaP_APK44) [5], K45 (phage vB_AbaM_B9) [8], K48 (phage vB_AbaP_APK48) [5], K87 (phage vB_AbaP_APK87) [5], K89 (phage vB_AbaP_APK89) [5], K91 (phage AP22) [13], K93 (phages vB_AbaP_APK2 and vB_AbaP_APK93) [5], and K116 (phage vB_AbaP_APK116) [5] capsular types of *A. baumannii* have been isolated and described. Genomes of all phages listed above encode only one tailspike depolymerase (TSD) specific to a certain K type or two K types with similar structures (for example K2/K93 in the case of phage vB_AbaP_APK2). 

In this study, we present a characterization of the novel bacteriophage TaPaz, the first reported virus specific to *A. baumannii* strain of the K47 capsular type. Genes encoding two TSDs that presumably allow TaPaz to infect two different K types were identified in the phage genome. For one of the TSDs responsible for the specific interaction of the phage with *A. baumannii* K47, the mechanism of specific CPS cleavage was established. The data on the characterization of phage TaPaz and encoding in its genome TSDs contributes to enhancing knowledge of the diversity of *A. baumannii* viruses and the mechanisms of their interaction with different *A. baumannii* K types.

## 2. Materials and Methods

### 2.1. Phage Isolation, Propagation, and Purification

Bacteriohage TaPaz was isolated from a sewage sample (collected in 2017, Moscow, Russia), in accordance with a previously reported procedure [12]. The sample was cleared by low-speed centrifugation (7000 G for 30 min), the supernatants supplemented with LB medium were incubated for 16–18 h in the presence of growing *A. baumannii* strains belonging to different capsular types at 37 °C, and then chloroform was added. Bacterial debris was pelleted by centrifugation at 7000 G for 30 min, followed by filtration of the supernatants through 1.20 and 0.45-µm-pore-size membrane filters, Millex-GV (Millipore, Cork, Ireland). The purified filtrates were concentrated by ultracentrifugation at 85,000 G at 4 °C for 2 h. The spot test method, as well as plaque assay [14], was used to screen for the presence of lytic phage activity in the resultant concentrated preparations. The plates were incubated overnight at 37 °C and examined for zones of lysis or plaque formation. 

Single plaques formed on the lawn of sensitive *A. baumannii* strain were picked up in the SM buffer (10 mM Tris-HCl, pH 7.5, 10 mM MgSO_4_, and 100 mM NaCl) and replated three times to obtain pure phage stock. 

*A. baumannii* strain NIPH601 (K47 capsular type) used as the bacterial host for phage isolation and propagation was kindly provided by Dr. Alexandr Nemec (Centre for Epidemiology and Microbiology, National Institute of Public Health, Czech Republic). The phage was propagated using liquid culture of *A. baumannii* NIPH601 (OD600 of 0.3) at the multiplicity of infection (MOI) of 0.1. The incubation was performed at 37 °C until lysis, and then chloroform was added. Bacterial debris was pelleted by centrifugation at 7000 G for 30 min. The phage preparation was purified by cesium chloride density-gradient centrifugation at 100,000 G (Beckman SW50.1 Ti rotor, Beckman Coulter Inc., Brea, CA, USA) for 24 h [15].

### 2.2. Phage Host Specificity Determination

For phage host specificity determination, a collection of various *A. baumannii* strains with defined CPS structure (listed in Table S1), kindly provided by the members of research groups from different countries (c.f. Acknowledgements), was used. In total, the host specificity of the phage was tested against *A. baumannii* strains belonging to 56 different K types using the double-layer method [14]. For this, 300 µL of *A. baumannii* bacterial cultures grown in LB medium at 37 °C to OD_600_ of 0.3 were mixed with 4 mL of soft agar (LB broth supplemented with 0.6% agarose). The mixture was plated onto the nutrient agar. Then, the phage suspensions (~10^9^ plaque-forming units (PFU) per mL) or purified recombinant depolymerase, and their several 2- and 10-fold dilutions, were spotted on the soft agar lawns and incubated at 37 °C for 18–24 h.

### 2.3. Phage Adsorption and One-Step Growth Experiments

For adsorption assay, exponentially grown *A. baumannii* NIPH601 bacterial cells were mixed with the phages (MOI = 0.001) and incubated at room temperature. A volume of 100 µL of samples was taken in 0, 1, 2, 3, 4, 5, 8, 10, and 15 min and then mixed with 850 µL of SM buffer supplemented with 50 µL of chloroform. After centrifugation, the supernatants were titrated for further determination of unabsorbed phages by the plaque assay method [14] at different time intervals. The adsorption constant was calculated according to the study by Adams [14] for a period of 5 min.

For the one-step growth experiments, 20 mL of host bacterial cells (OD_600_ of 0.3) was harvested by centrifugation (3500 G, 10 min, 4 °C) and resuspended in 1 mL LB broth. Bacterial cells were infected with the phage at MOI of 0.01. The bacteriophage was allowed to adsorb for 5 min at 37 °C. Then, the mixture was centrifuged at 13,000 G for 2 min to remove unabsorbed phage particles, and the pellet was resuspended in 20 mL of LB broth. Samples were taken at 10-min intervals for 2 h incubation at 37 °C and immediately titrated. The procedures were repeated three times.

### 2.4. Electron Microscopy

The phage was examined by negative contrast electron microscopy, using the following procedure. 3 µL of purified and concentrated phage preparation was applied to the carbon-coated 400 mesh copper grids and subjected to glow-discharge using the Emitech K100× apparatus (Quorum Technologies, Laughton, UK). Grids were then negatively stained with 1% uranyl acetate for 30 s, air-dried, and analyzed using a JEOL JEM-2100 200kV transmission electron microscope. Images of negatively stained phage particles were taken with a Gatan Ultrascan 1000XP CCD camera (14 mkm pixels) and Gatan Digital Micrograph software with the following parameters: 30,000× magnification, 0.5–1 µm defocus, 40 µm objective aperture, 2k × 2k pixel size unbinned image size, 3.4 angstrom pixel size [16]. At least 30 electronic phage images were used for the phage morphology determination.

### 2.5. DNA Isolation and Sequencing

Phage genomic DNA was isolated from concentrated and purified high titer phage stocks by incubation in 0.5% SDS, 20 mM EDTA, and 50 µg/mL proteinase K at 56 °C for 1–3 h. The DNA was extracted with phenol-chloroform and then precipitated with ethanol supplemented with sodium acetate [15]. Genome sequencing was performed on the MiSeq platform using a Nextera DNA library preparation kit (Illumina, San Diego, CA, USA). The generated reads were assembled de novo into single contig using SPAdes v. 3.13 [17] with default parameters. 

### 2.6. Phage Genome Analysis

Potential open reading frames (ORFs) were identified with the RAST automated annotation engine [18] and then manually inspected. Predicted proteins were searched against the NR (non-redundant) database of the NCBI [19] and HHpred profile-profile search [20]. ORFs were also compared against Virulence Factors of Pathogenic Bacteria (VFDB) [21]. The presence of tRNAs in the genome sequence was determined using ARAGORN [22]. Average nucleotide identity (ANI) was calculated using the OrthoANIu tool [23]. Genome visualization and comparison were made with Easyfig [24]. Phylogenetic analysis was performed by using the amino acid sequences of major capsid proteins, large terminase subunits, portal proteins and tail tape measure proteins encoded in related myoviruses deposited in the NCBI GenBank database. Considering that the large terminase subunit of phage TaPaz was determined to contain an intein splicing domain, the amino acid sequence without fragment from 35 to 388 residues of TaPaz_gp49 was used for the phylogenetic tree construction. Gene products in other phage genomes annotated as “hypothetical proteins” were considered as known genes if their pairwise identity with known homologous was more than 50%. The alignments were made with MAFFT with L-INS-i settings [25]. The best protein substitution model was found with MEGAX [26]. The phylogenetic tree was constructed with RAxML by rapid bootstrapping (bootstrap 1000) with GAMMA LG F protein model [27].

### 2.7. Nucleotide Sequence Accession Number

The genome sequence of phage TaPaz was deposited to GenBank under accession number MZ043613.

### 2.8. Cloning, Expression, and Purification of the Recombinant Depolymerase

The DNA fragments corresponding to the deletion mutants lacking N-terminal domain of phage TSDs TaPaz_gp78 and TaPaz_gp79 were amplified by PCR, using oligonucleotide primers ataGGATCCagtgctgcaccttcccaca and ataCTCGAGttatattgatgaaagaataaacatg (for TSD TaPaz_gp78), ataGGATCCtctaacgatttaataccacaact and ataCTCGAGttagttttctaacatgagtcga (for TSD TaPaz_gp79), and cloned into the pTSL plasmid (GenBank accession KU314761) [28].

Expression vectors were transformed into chemically competent *Escherichia coli* BL21 (DE3) cells. Protein expression was performed in LB medium supplemented with ampicillin at 100 mg/L. Transformed cells were grown at 37 °C until the optical density reached the value of 0.4 at 600 nm. The medium was cooled to the temperature of 16 °C followed by expression induction by the addition of isopropyl-1-thio-β-d-galactopyranoside (IPTG) to a final concentration of 1 mM. After further incubation at 16 °C overnight (approximately 16 h), the cells were harvested by centrifugation at 3700 G for 20 min, 4 °C. The cell pellets were resuspended in 1/50th of the original cell volume in buffer A (20 mM Tris pH 8.0, 0.5 M NaCl, 20 mM imidazole) complemented with 1 mg/mL lysozyme and then lysed by sonication. The cell debris was removed by centrifugation at 16,000 G for 30 min, 4 °C. The supernatants were loaded onto nickel Ni^2+^-charged 5 mL GE HisTrap columns (GE Healthcare Life Sciences, Chicago, IL, USA) equilibrated with buffer A containing 20 mM imidazole, and eluted with a 20–500 mM imidazole linear gradient in buffer A. The fractions containing the target proteins were pulled together and set up at 4 °C for the His-tag overnight digestion with TEV-protease at a protease/protein ratio of 1/100 (*w*/*w*). This reaction mixture was simultaneously dialyzed against 20 mM Tris pH 8.0, 200 mM NaCl, 0.5 mM DTT buffer resulting into His-SlyD expression tag removal. Protein samples after digestion were applied to the His-Trap column as before. A concentrated with Sartorius ultrafiltration devices (molecular weight cutoff of 10,000, Sartorius, Gottingen, Germany) flow-through was applied to a Superdex 200 Hiload 16/60 column (GE Healthcare Life Sciences) pre-equilibrated in 20 mM Tris-HCl, pH 7.5, and 150 mM NaCl (buffer B). The final protein samples were concentrated with Sartorius ultrafiltration devices and stored in the same buffer at 4 °C. The protein concentration was determined using the Qubit Protein Assay Kit and the Qubit 4.0 fluorometer (Invitrogen, Carlsvad, CA, USA).

### 2.9. Phage Infection Inhibition Assay

TaPaz-TSD infection inhibition assay was performed according to the published procedure [29]. A titer of 2.5 × 10^6^ PFU/mL for the phage was chosen for the competition experiments. *A. baumannii* NIPH601 was grown in LB medium at 37 °C to an OD_600_ of 0.3. Then, TSD TaPaz_gp79 was added to a 100-μL aliquot of the cell culture to a final concentration of 0.5 mg/mL and incubated for 20 min at 37 °C. One-hundred-microliter aliquots of the *A. baumannii* host cells without anything and with TSD TaPaz_gp78 and bovine serum albumin (BSA) to a final concentration of 0.5 mg/mL incubated for 20 min at 37 °C served as controls. After the incubation, several dilutions of phage TaPaz and 4 mL of soft agar were added to the mixtures and plated onto the nutrient agar. The plates were incubated overnight at 37 °C and assayed for the number of lysis plaques. The experiment was performed in triplicate. The GraphPad Prism software (GraphPad Software, Inc., La Jolla, CA, USA) was used for statistical analysis and graphical presentation of the results.

### 2.10. Isolation and Purification of the CPS

*A. baumannii* NIPH601 was cultivated in 2TY media (16 g Bacto Tryptone, 10 g Bacto Yeast Extract, and 5 g NaCl, adjusted to 1 L with distilled H_2_O) for 16 h. Bacterial cells were harvested by centrifugation (10000 G, 20 min), washed with phosphate-buffered saline, suspended in aqueous 70% acetone, precipitated, and dried on air.

A K47 CPS sample was isolated by extraction of bacterial cells with 45% aqueous phenol for 30 min at 65–68° C [30] and purified as described [31]. 

### 2.11. Cleavage of the CPS with TSD TaPaz_gp79 

A purified K47 CPS sample (24 mg) was solubilized in the 20 mM Tris-HCl pH 7.5 buffer and 500 µg of recombinant TSD TaPaz_gp79 was added for digestion. The reaction mixture was incubated at 37 °C overnight. 

CPS digestion products were fractionated by gel-permeation chromatography on a XK 16/100 column (110 cm × 16 mm, gel layer 90 cm) of Sephadex G-25 (GE Healthcare, UK) in 1% acetic acid at a flow rate of 0.5 mL/min monitored using a differential refractometer (Knauer, Berlin, Germany). Three fractions corresponding to a monomer, a dimer, and a higher molecular-mass product(s) were obtained in yields 3.9, 11.2, and 4.2 mg, respectively.

### 2.12. NMR Spectroscopy

Samples were deuterium-exchanged by freeze-drying from 99.9% D_2_O and then examined as the solution in 99.95% D_2_O. NMR spectra were recorded on a Bruker Avance II 600 MHz spectrometer (Bruker, Ballerica, MA, USA) at 30–60 °C. Sodium 3-trimethylsilylpropanoate-2,2,3,3-d_4_ (δ_H_ 0, δ_C_ −1.6) was used as internal reference for calibration. Two-dimensional ^1^H-^1^H correlation spectroscopy (COSY), ^1^H ^1^H total correlation spectroscopy (TOCSY), ^1^H-^1^H rotating-frame nuclear Overhauser effect spectroscopy (ROESY), ^1^H-^13^C heteronuclear single-quantum coherence (HSQC), and ^1^H-^13^C heteronuclear multiple-bond correlation (HMBC) experiments were performed using standard Bruker software and were used for assignment of ^1^H and ^13^C NMR chemical shifts [32]. Bruker TopSpin 2.1 program was used to acquire and process the NMR data. A MLEV-17 spin-lock time of 60 ms and a mixing time of 200 ms were used in TOCSY and ROESY experiments, respectively. A 60-ms delay was used for the evolution of long-range couplings to optimize the ^1^H,^13^C HMBC experiment for coupling constant *J*_H,C_ 8 Hz.

### 2.13. Mass Spectrometry

High-resolution electrospray ionization mass spectrometry (HR ESI MS) [33] was performed in the positive ion mode using a micrOTOF II instrument (Bruker Daltonics). Oligosaccharide samples (~50 ng/µL) were dissolved in a 1:1 (*v*/*v*) water acetonitrile mixture and injected with a syringe at a flow rate of 3 µL/min. Capillary entrance voltage was set at −4500 V, and the interface temperature at 180 °C. Nitrogen was used as the drying gas. Mass range was from *m/z* 50 to 3000. Internal calibration was done with ESI Calibrant Solution (Agilent).

## 3. Results and Discussion

### 3.1. Phage Isolation, Morphology, Host Specificity, and Infection Parameters

**Figure 1:**
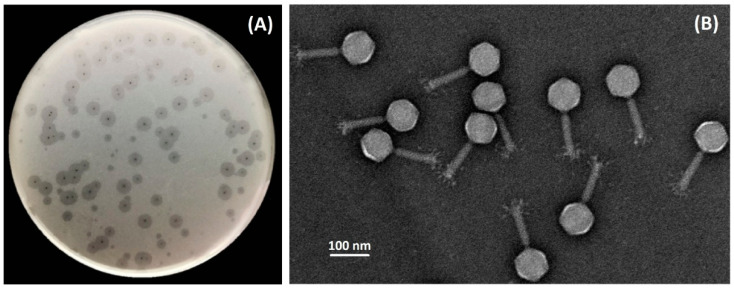
Morphological characteristics of phage TaPaz. (**A**) Phage plaques with opaque haloes on *A. baumannii* NIPH601. (**B**) Transmission electron micrographs of the phage particles. Staining with 1% uranyl acetate. The scale bar is 100 nm.

Bacteriophage TaPaz was isolated on the bacterial lawn of *A. baumannii* NIPH601 belonging to K47 capsular type from a sewage sample using an enrichment procedure. On the lawn of the host strain phage TaPaz produces small clear plaques surrounded by haloes (Figure 1A) indicating the presence of phage structural depolymerase degrading the K47 CPS layer. Transmission electron microscopy of negatively stained phage particles revealed that the phage has an icosahedral head of 85 ± 3 nm in diameter and a contractile tail of 110 ± 5  nm in length with distinguishable structural appendages (Figure 1B). The phage can be assigned morphologically to the family *Myoviridae*. 

The host specificity of TaPaz was tested against a collection of *A. baumannii* strains with biochemically characterized CPS structures belonging to 56 different K types used in our previous study [5] (Table S1). It was found that TaPaz was highly specific as the other depolymerase-carrying *A. baumannii* phages and was cable to infect only a strain with a certain CPS structure, namely *A. baumannii* NIPH601 of K47 capsular type. 

**Figure 2:**
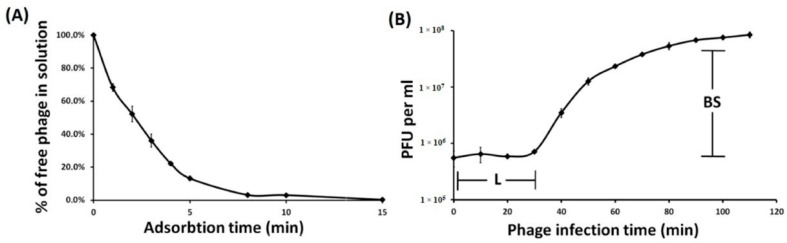
Infection analysis of phage TaPaz. Adsorption assay (**A**) and one-step growth curve (**B**) of phage TaPaz on *A. baumannii* NIPH601 with the indication of estimated burst size (BS) and latent period (L). Results are the means and standard deviations from three independent experiments. PFU: plaque-forming units.

The infection process was determined by estimating adsorption efficiency and phage infection parameters in TaPaz-*A. baumannii* NIPH601 system. It was determined that 80% of phage particles adsorbed to *A. baumannii* NIPH601 cells within 4 min, and more than 95% within 15 min (Figure 2A). Phage TaPaz exhibited adsorption constant of 4.04 × 10^−8^ mL/min for the host strain for a period of 5 min. 

To identify the different phases of the phage infection process, one-step growth experiments were performed (Figure 2B). The latent period for TaPaz was about 30 min, and the burst size was approximately 110 particles per infected cell.

### 3.2. Phage Genome Organization and Comparison 

Phage TaPaz has a 93,703-bp linear double-stranded DNA genome with a G + C content of 32.64%, which is close to phage vB_AbaM_B9 (32.57%) [8] but lower than almost all other *A. baumannii* phages and *A. baumannii* strains (approximate average values are 38.94–39.4% [34]).

The genome was found to contain 178 putative open reading frames (ORFs), 111 of which are located on the forward strand, and 67 are located on the reverse strand. Putative functions were assigned to 43 of 178 (24.2%) products of predicted ORFs. The other 135 ORFs (75.8%) encoded hypothetical proteins with unknown functions or did not match any characterized functional proteins. The search for tRNA sequences using ARAGORN [22] revealed a region encoding one tRNA for Arg.

**Figure 3:**
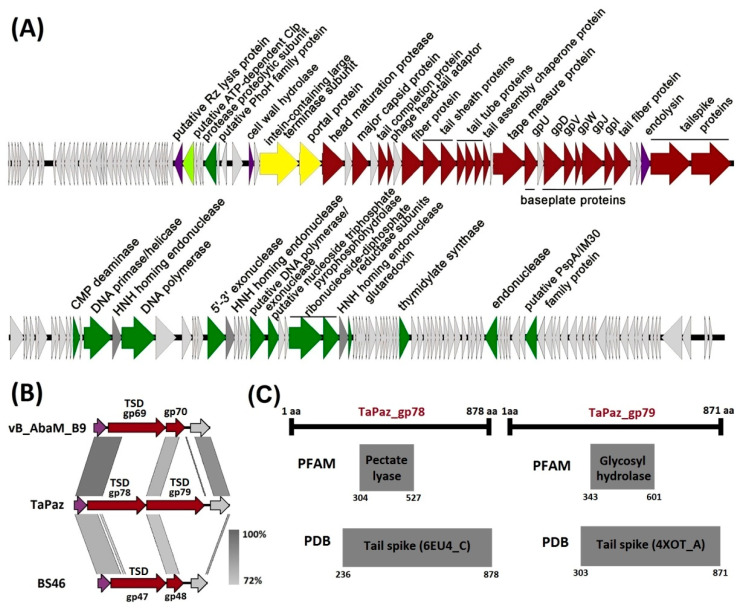
(**A**) Genetic map of phage TaPaz. The ORFs are represented as colored arrows according to functional predictions: green, genes coding for enzymes of nucleotide metabolism and proteins involved in DNA replication and repair; light green, gene for protein with protease activity; yellow, genes for packaging of DNA; red, structural protein and phage assembly protein genes; violet, lysis protein genes; dark grey, HNH homing endonuclease genes; light gray, hypothetical protein genes. The direction of an arrow shows the direction of transcription. (**B**) Comparison of regions of vB_AbaM_B9, TaPaz, and BS46 genomes that contain genes encoding tailspikes: violet, genes coding endolysins; red, structural protein genes; light gray, hypothetical protein genes; tailspike depolymerases are marked as TSD. (**C**) HHpred-detected similarities between TSDs TaPaz_gp78 and TaPaz_gp79 and proteins from the Pfam and PDB databases.

The genome organization of phage TaPaz is shown in Figure 3A. The proteins encoded in the phage genome can be divided into several functional groups: phage assembly and structural proteins (major capsid protein, tail sheath and tail tube proteins, baseplate proteins, tail fiber proteins, tailspike proteins, etc.), proteins involved in nucleotide metabolism, DNA replication and repair (CMP deaminase, DNA primase/helicase, DNA polymerase, 5′-3′ exonuclease, alpha and beta subunits of ribonucleoside-diphosphate reductase, thymidylate synthase, etc.), packaging of DNA into the capsid (large terminase subunit, portal protein) and proteins involved in the bacterial cell lysis (cell wall hydrolase, endolysin, etc.) (Figure 3A). 

It should be noted that the structural module of the TaPaz genome contains genes encoding two tailspike proteins (TaPaz_gp78 and TaPaz_gp79) and two fiber proteins (TaPaz_gp58 and TaPaz_gp74), indicating a complex organized adsorption apparatus of the phage (Figure 3A). 

Despite modular organization, genes encoding proteins involved in disruption or lysis of the bacterial cell are distributed throughout the TaPaz genome, for example, before the genes responsible for the packaging of DNA into the capsid and within the structural module before the genes encoding tailspikes (Figure 3A). 

It is noteworthy that the large terminase subunit TaPaz_gp49 was predicted by BLASTp and HHpred to contain an intein domain (35–388 residues within TaPaz_gp49). Inteins or protein introns are self-splicing mobile genetic elements found in all domains of life [35]. It was previously mentioned that phage terminases are a common target for intein insertions [36,37]. ORFs encoding three putative HNH homing endonucleases (TaPaz_gp94, gp102, and gp112) were also found in the nucleotide metabolism and DNA replication genome module. 

No genes encoding toxins, factors responsible for antibiotic resistance or products related to lysogeny were identified in the TaPaz genome.

Phage TaPaz shares the highest similarity at the DNA level with *Acinetobacter* myovirus vB_AbaM_B9 [8] (GenBank accession number: MH133207, the genome coverage obtained to an *E*-value of 0.0 was 60% with an identity of 96% according to BLAST) isolated in Portugal. According to ANI calculations, TaPaz demonstrates 90.58% identity with vB_AbaM_B9, therefore these phages should be considered as separate species. Also, the phage shares DNA similarity with *Acinetobacter* myovirus BS46 [11] (GenBank accession number: MN276049, 79.47% ANI identity with TaPaz), *Edwardsiella* phage vB_EtaM_ET-ABTNL-9 (GenBank accession number: MN399336, 63.59% ANI identity with TaPaz), and *Vibrio* phage vB_VhaM_VH-8 (GenBank accession number: MN497415, 63.09% ANI identity with TaPaz). 

**Figure 4:**
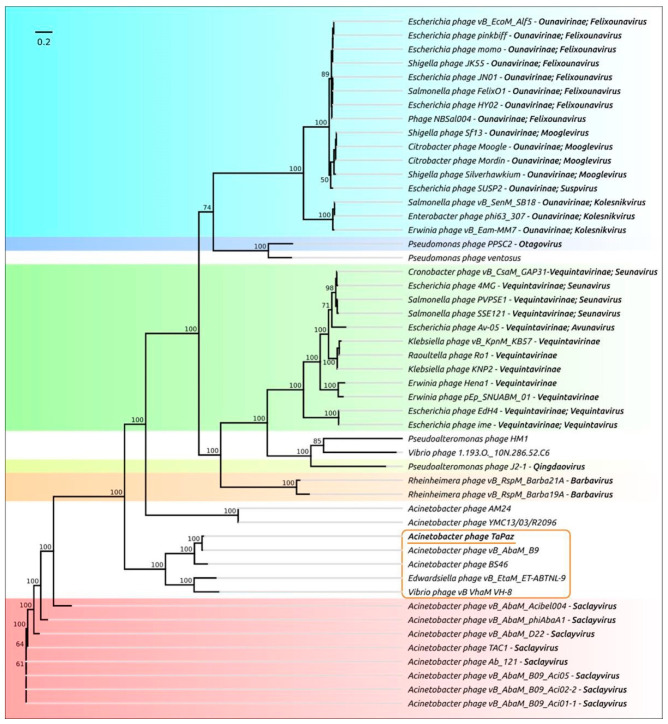
Best-scoring phylogenetic tree based on concatenated sequences of a major capsid protein, a large subunit of terminase, a portal protein and a tail tape measure protein from the genomes of 50 phages. Taxonomic classification is taken from ICTV (International Committee on Taxonomy of Viruses) and NCBI sequence attributes and is shown to the right of the organism’s name. Bootstrap support values are shown near their branch as a percentage of 1000 replicates. The scale bar shows 0.2 estimated substitutions per site and the tree was unrooted.

To clarify the taxonomic position of phage TaPaz, a phylogenetic tree with concatenated alignments of amino acid sequences of four genes (encoding major capsid protein, terminase large subunit, portal protein, and tail tape measure protein) found with BLAST search was generated (Figure 4). The results of the phylogenetic analysis showed that phage TaPaz was most closely related to phage vB_AbaM_B9 and together with *Acinetobacter* myovirus BS46, *Edwardsiella* phage vB_EtaM_ET-ABTNL-9, and *Vibrio* phage vB_VhaM_VH-8 formed a distinct monophyletic branch. *A. baumannii* myoviruses of *Saclayvirus* genus and unclassified *Acinetobacter* myoviruses AM24 and YMC13/03/R2096 appear to represent the closest groups. The representatives of *Barbavirus* and *Qingdaovirus* genera and *Vequintavirinae* and *Ounavirinae* subfamilies formed more distantly related groups.

### 3.3. Phage TaPaz Structural Tailspike Depolymerases

On the example of several previously characterized *A. baumannii* phages, it was shown that TSDs encoding in their genomes are responsible for the ability of bacterial viruses to infect the strains with certain CPS structures [5,6,7,8,9,10,12]. Genes encoding two different TSDs that presumably allow TaPaz to infect the representatives of two various *Acinetobacter* K types were identified in the phage genome. Both TSDs TaPaz_gp78 and TaPaz_gp79 were formed by single proteins encoded by the genes located at the end of the structural module of the TaPaz genome (Figure 3A). 

BLASTp analysis revealed that the closest homologs of TSDs TaPaz_gp78 and TaPaz_gp79 were tail fiber domain-containing protein of *Acinetobacter nosocomialis* (WP_151789757, the coverage obtained to an E-value of 0.0 was 73% with an identity of 74.88%) and hypothetical protein of *A. baumannii* (WP_038405668, the coverage obtained to an *E*-value of 0.0 was 74% with an identity of 50.38%), respectively. However, in both cases, there was no homology between N-terminal parts of TaPaz_gp78 and TaPaz_gp79 amino acid sequences and N-terminal parts of sequences of these proteins at all. On the contrary, the N-terminal part of TSD TaPaz_gp78 shares a high level of similarity with N-terminal parts of tailspike protein gp69 of *Acinetobacter* phage vB_AbaM_B9 (AWD93192, the coverage obtained to an E-value of 3e^−124^ was 33% with an identity of 76.90%) and tailspike protein gp47 of *Acinetobacter* phage BS46 (QEP53229, the coverage obtained to an *E*-value of 9e^−101^ was 37% with an identity of 56.14%) (Figure 3B). N-terminal part of TSD TaPaz_gp79 shares a high level of similarity with gp70 of *Acinetobacter* phage vB_AbaM_B9 (AWD93215, the coverage obtained to an *E*-value of 2e^−101^ was 24% with an identity of 76.39%) and putative tail fiber protein gp48 of *Acinetobacter* phage BS46 (QEP53230, the coverage obtained to an *E*-value of 4e^−91^ was 24% with an identity of 72.17%) (Figure 3B). Thus, N-terminal parts of TSDs TaPaz_gp78 and TaPaz_gp79 responsible for the attachment of variable CPS-recognizing/degrading parts of the tailspikes to the phage particles were quite conservative within the group of phages (TaPaz, vB_AbaM_B9, and BS46) sharing the similar genome organization and the highest percentage of homology at the DNA level. While CPS-recognizing/degrading parts of TaPaz_gp78 and TaPaz_gp79 were highly homologous to different proteins encoded in *A. nosocomialis* and *A. baumannii* genomes. 

Interestingly, phage TaPaz encoded two different complete TSDs, whereas phage vB_AbaM_B9 as well as phage BS46 encoded only one complete depolymerase (gp69 and gp47, respectively) sharing homology with the N-terminal part of TaPaz_gp78. Gp70 (291 aa) of phage vB_AbaM_B9 and gp48 (256 aa) of phage BS46 presumably could be only the N-terminal parts of complete TSDs without CPS-recognizing/degrading parts (Figure 3B).

According to HHpred analysis the amino acid sequences of TaPaz_gp78 and TaPaz_gp79 had the pectate lyase 3 (PF12708.9; E-value of 1.5e^−9^) and Glyco_hydro_28 (PF00295.19; E-value of 1.3e^−11^) conserved Pfam motifs, respectively. Moreover, the predicted structures of TaPaz_gp78 and TaPaz_gp79 were similar to the structure of *A. baumannii* phage vB_AbaP_AS12 tailspike (PDB accession number 6EU4) and tailspike protein of *E. coli* bacteriophage HK620 (PDB accession number 4XOT), respectively (Figure 3C).

**Figure 5:**
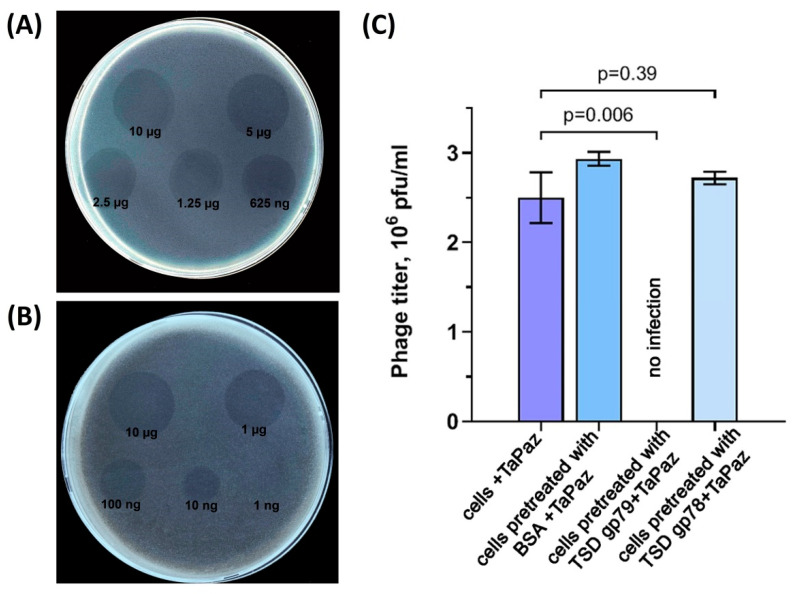
Spot tests with serial 2-fold (**A**) and 10-fold (**B**) titration of purified recombinant depolymerase TaPaz_gp79 on *A. baumannii* NIPH601 lawn after 12 h of incubation. (**C**) Phage TaPaz infection inhibition by TSD TaPaz_gp79. From left to right, phage titers observed on the bacterial lawns after the treatment of *A. baumannii* NIPH601 cells with phage TaPaz only, after cell cultures preincubated with BSA (as a negative control), purified specific TSD TaPaz_gp79 and purified not specific TaPaz_gp78 (as a negative control) followed by phage TaPaz treatment.

TaPaz_gp78 and gp79 are 878- and 871-amino-acid proteins with predicted molecular weights of 95.9 kDa and 96.2 kDa, respectively. Deletion mutants lacking the N-terminal domains of the TaPaz tailspikes were cloned, expressed and purified by immobilized metal ion affinity chromatography, followed by gel permeation chromatography. The spectra of depolymerase activities of recombinant proteins were tested against a panel of *A. baumannii* strains belonging to 56 different K types (Table S1). Purified recombinant depolymerase TaPaz_gp79 was found to be highly specific and formed opaque halo only on the bacterial lawn of *A. baumannii* NIPH601, meaning that this depolymerase effectively degraded K47 CPS of the host strain. Serial 2-fold and 10-fold titrations of the purified recombinant depolymerase TaPaz_gp79 on the bacterial lawn of *A. baumannii* NIPH601, after 12 h of incubation, are presented in Figure 5A,B. 

Unfortunately, we were unable to determine the specificity of purified TSD TaPaz_gp78 using a collection of *A. baumannii* strains including representatives of 56 K types. Considering the fact that nowadays more than 140 KL variants (chromosomal capsule loci, K loci or KL) have been bioinformatically predicted, this means that TSD TaPaz_gp78 quite possibly could specifically interact with a CPS of one of those K types that have not been tested in this study. Besides, the closest homolog of TaPaz_gp78 was found to be a protein encoded in the *A. nosocomialis* genome. Thus, TSD TaPaz_gp78 could possibly interact with CPS produced by not *A. baumannii*, but *A. nosocomialis* and TaPaz could be considered as multi-host phage. These statements require further experimental confirmation.

To show that TSD TaPaz_gp79 is responsible for the initial step of the phage TaPaz-K47 bacterial cell interaction, the competition experiments have been performed (Figure 5C). For this, *A. baumannii* NIPH601 bacterial culture preincubated with purified TSD TaPaz_gp79 was mixed with several phage TaPaz dilutions and plated on agar plates. In negative-control experiments host bacterial cells were pretreated with BSA and TSD TaPaz_gp78 which is not specific to K47 CPS. After overnight incubation, phage titer was measured. It was shown that TSD TaPaz_gp79 fully blocked the phage infection, whereas coincubation of host bacterial cells with BSA and TSD TaPaz_gp78 showed no significant differences in phage titers. The results obtained from competition experiments are presented in Figure 5C.

### 3.4. Cleavage of the A. baumannii NIPH 601 CPS by TSD TaPaz_gp79

To elucidate the mechanism of action of TSD TaPaz_gp79, K47 CPS degradation by the purified recombinant protein was performed.

**Figure 6:**
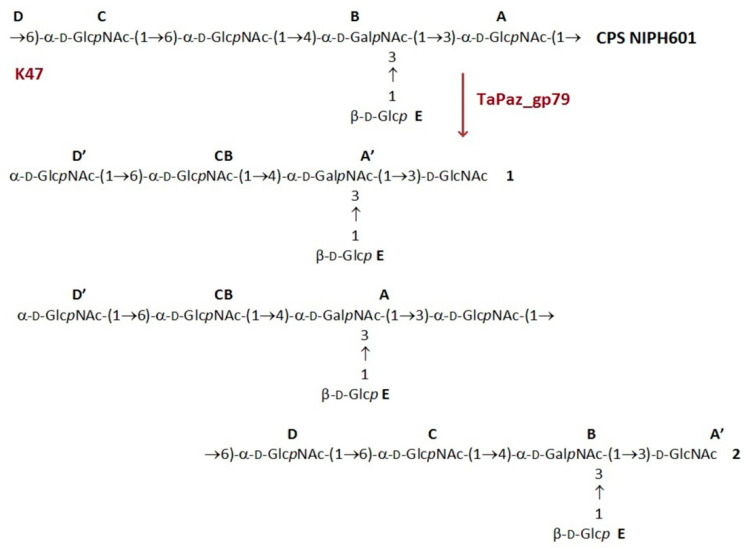
Cleavage of the K47 CPS of *A. baumannii* NIPH601 with TSD TaPaz_gp79 giving rise to oligosaccharides **1** and **2** corresponding to a monomer and a dimer of the K unit, respectively.

The structure of the K47 CPS from the host *A. baumannii* strain belonging to the K47 capsular type has been established earlier [38]. The CPS was determined to build up of pentasaccharide repeats (K units) containing one residue each of D-Glc (E) and D-GalNAc (B) and three residues of D-GlcNAc (A, C, D) (Figure 6).

The CPS was cleaved with recombinant TSD TaPaz_gp79, and oligosaccharide products were fractionated by Sephadex G-25 gel-permeation chromatography. The CPS gave oligosaccharides **1** and **2** as well as a minor higher-molecular mass product corresponding to a higher K unit oligomer(s). All isolated oligosaccharides had the same monosaccharide composition as the initial CPS.

Oligosaccharides **1** and **2** were studied by positive ion mode high-resolution electrospray ionization mass spectrometry (HR ESI MS) [32] and one- and two-dimensional ^1^H and ^13^C nuclear magnetic resonance (NMR) spectroscopy [33]. The mass spectra of **1** and **2** showed the major [M+Na]+ ion peaks at m/z 1015.3678 and 1989.7392 against the calculated m/z values of 1015.3701 and 1989.7404 for a monomer and a dimer of the K unit, respectively.

The ^1^H and ^13^C NMR spectra of the oligosaccharides **1** and **2** were fully assigned by two-dimensional shift-correlated experiments (^1^H-^1^H COSY, ^1^H-^1^H TOCSY, and ^1^H-^13^C HSQC) and compared with published data of the corresponding CPS. The ^13^C NMR chemical shifts of three from five monosaccharide residues (B, C, and E) were essentially the same in the CPS and the oligosaccharides, whereas those of two other residues (A’ and D’) were different. The reducing end in **1** is occupied by D-GlcNAc (residue A’), which showed in the ^13^C NMR spectrum C-1 signals at d 92.2 and 95.8 characteristic for its a- and b-anomeric forms, respectively [39]. The ^13^C NMR chemical shifts of a-GlcNAc that is 6-substituted in the CPS (residue D, C-6 resonance at d 66.2), are typical of the corresponding nonsubstituted residue in **1** (residue D’, C-6 resonance at d 61.8) [39]. The chemical shifts of residue A’ and D’ in **2** were similar to those in **1**. These data defined the structures of pentasaccharide **1** and decasaccharide **2** shown in Figure 6.

To sum up, TSD TaPaz_gp79 cleaved specifically the K47 CPS of *A. baumannii* NIPH601 by the hydrolytic mechanism by the α1 → 6-linkage between two D-Glc*p*NAc residues (A and D) to give oligosaccharides **1** and **2** corresponding to a monomer and a dimer of the K unit, respectively.

## 4. Conclusions

In this study, we present a characterization of the novel bacteriophage TaPaz belonging to the family *Myoviridae*. This is the first reported *A. baumannii* virus carrying two different TSDs, one of which is specific to *A. baumannii* strain of the K47 capsular type. Analysis of oligosaccharide products obtained by degradation of the K47 CPS by the recombinant depolymerase TaPaz_gp79 showed that the TSD was a specific glycosidase that cleaved the CPS by the hydrolytic mechanism, to give a monomer and an oligomer of the K unit. The comprehensive characterization of new lytic *A. baumannii* phages and their receptor-binding proteins expand our knowledge of strategies of virus–bacteria interaction. 

## Data Availability

The genome sequence of phage TaPaz is available in Genbank at accession number MZ043613.

## Supplementary Materials

The following are available online at https://www.mdpi.com/article/10.3390/v13060978/s1, Table S1: *Acinetobacter baumannii* strains used in this study for phage and TSDs specificities determination.
