# Supplementary material for: Novel Acinetobacter baumannii Myovirus TaPaz Encoding Two Tailspike Depolymerases: Characterization and Host-Recognition Strategy
Source: Viruses. 2021 May 25;13(6):978. doi: 10.3390/v13060978 (PMC8228797; doi:10.3390/v13060978)
Supplement: Supplementary file 1 [file viruses-13-00978-s001.zip › viruses-1223114-supplementary.pdf]

Supplementary Materials

Table S1. ***Acinetobacter baumannii*** **strains used in this study for phage and TSDs specificities determination**

K-type	*A. baumannii* strains with confirmed CPS structure	KL GenBank accession number or coordinates within whole genome shotgun sequences
1	AYE	CU459141 (base position range: 3863316-3834156)
2	ACICU	CP000863 (base position range: 85537- 29488)
3/22[a]	ATCC17978/LUH5537	CP000521 (base position range: 56835-79908)/ KC526920
6	RBH4	KF130871
7	LUH5533	KC526894
8	BAL097	KX712116
9	B05	MK331712
11	LUH5545	KC526904
15	LUH5554	KC526900
16	D4	MF522813
17	G7	KC118541
19	28	KU215659
20	A388	JQ684178
21	G21	MG231275
24	RCH51	KX756650
25	AB5075	BK008886
27	4190	KT266827
30	NIPH190	MN166189
32	LUH5549	KC526897
33	NIPH67	MN166195
35	LUH5535	KC526896
37	NIPH146	APOU01000009 (base position range: 32574-53092)
42	LUH5550	KC526903
43	LUH5544	KC526905
44	NIPH70	APRC01000043 (base position range: 97989-129118)
45	NIPH201	MN166190
46	NIPH329	MK609549
47	NIPH601	MN166193
48	NIPH615	MN166191
51	WM98b	MN148384
52	LUH5546	KC526899
53	D23	MH190222
54	RCH52	MG867726

55	BAL204	MN148381
57	BAL212	KY434631
58	BAL114	KT359617
61	NL4	to be registered
73	SGH0703	MF362178
74	BAL309	MN148383
80	LUH3712	KC526914
81	LUH3713	KC526916
82	LUH5534	KC526908
83	LUH5538	KC526898
84	LUH5540	KC526902
85	LUH5543	KC526913
87	LUH5547	KC526918
88	LUH5548	KC526910
89	LUH5552	KC526919
90	LUH5553	KC526917
91	1053	KM402814
92	B8300	CP021347 (base position range: 1420707-1451977)
93	B11911	BK010902
116	MAR-303	MK399425
125	MAR13-1452	MH306195
128	KZ-1093	MK399428

[a] These KL clusters are closely related, though contain a little difference at the nucleotide level, but produce CPSs with identical structures.
